# Integrated mRNA-miRNA Transcriptome Analysis Reveals the Molecular Mechanism of Tibetan Sheep Rumen Epithelium Adaptation to High Altitude

**DOI:** 10.3390/ani16111650

**Published:** 2026-05-28

**Authors:** Lei Wang, Wei Huang, Yuzhu Sha, Yanyu He, Pengyang Shao, Qianling Chen, Yapeng He, Jiangfeng Fan, Xiu Liu, Wenhui Du

**Affiliations:** 1 College of Veterinary Medicine, Gansu Agricultural University, Lanzhou 730070, China; 13519616029@163.com (L.W.); fanjiangfeng2008@163.com (J.F.); 2 Zhangye Livestock Breeding and Improvement Station, Zhangye 734000, China; 3 College of Animal Science and Technology, Gansu Agricultural University, Lanzhou 730070, China; 18294737108@163.com (W.H.); shayz@st.gsau.edu.cn (Y.S.); shaopengyang666@163.com (P.S.); chenqianling223@163.com (Q.C.); 18894448066@163.com (Y.H.); 4 School of Fundamental Sciences, Massey University, Palmerston North 4410, New Zealand; y.he@massey.ac.nz; 5 Gansu Provincial Animal Husbandry Technology Extension Station, Lanzhou 730050, China

**Keywords:** Tibetan sheep, altitude, rumen epithelium, mRNA, miRNA, transcriptome

## Abstract

The rumen epithelium of Tibetan sheep plays a critical role in energy metabolism and immune defense; however, its post-transcriptional regulatory mechanisms under high-altitude hypoxia stress remain unclear. In this study, we employed integrated mRNA and miRNA transcriptome sequencing to analyze the adaptive strategies of the rumen epithelium in Tibetan sheep at different altitudes. A total of 2183 differentially expressed genes (DEGs) and 135 differentially expressed miRNAs (DEmiRNAs) were identified. Functional enrichment analysis revealed that DEGs and their target genes were significantly enriched in immune-related pathways such as the NF-κB signaling pathway and cytokine–cytokine receptor interaction, as well as metabolic pathways including oxidative phosphorylation and branched-chain amino acid degradation. Integrated network analysis highlighted key regulatory pairs, including *oar-miR-370-3p* targeting *PCK2* and *IL1R2*, and *novel-miR-781* regulating *PIK3R5*, suggesting coordinated modulation between mitochondrial homeostasis and immune responses. Specifically, the upregulation of immune genes (*CCL19*, *MADCAM1*) and heat shock proteins at TS4500m indicates enhanced mucosal immunity and stress tolerance, while altered expression of metabolic genes reflects a shift in energy substrate utilization. These findings elucidate a complex mRNA-miRNA regulatory network that enables Tibetan sheep to maintain rumen epithelial integrity and energy balance under extreme high-altitude conditions, providing novel insights into the molecular basis of hypoxia adaptation in ruminants.

## Simple Summary

Tibetan sheep living on the Qinghai–Tibet Plateau have evolved strong adaptability to cold and low-oxygen environments, but the molecular mechanism by which their rumen epithelium copes with high-altitude stress remains unclear. This study aimed to reveal the adaptive regulatory mechanism using mRNA-miRNA transcriptome analysis. We identified many differentially expressed genes and microRNAs in the rumen epithelium of sheep at three altitudes. The results showed that with increasing altitude, Tibetan sheep enhance immune function and stress resistance by upregulating immune and heat shock genes, and maintain energy balance by adjusting metabolic pathways. Key miRNA-mRNA regulatory pairs were found to jointly control mitochondrial function and immune responses. These findings clarify the molecular mechanism of rumen epithelium adaptation to high altitude, providing a scientific basis for breeding livestock that can adapt well to plateau environments.

## 1. Introduction

The Qinghai–Tibet Plateau is known as the “Roof of the World”, with high cold and hypoxia as its dominant environmental characteristics. Tibetan sheep are an endemic and dominant livestock germplasm resource of the Qinghai–Tibet Plateau, characterized by cold tolerance, hypoxia resistance, roughage tolerance, and strong disease resistance, with unique biological mechanisms for high-altitude adaptation [1,2]. Genetic adaptation lies at the core of evolutionary biology research, and the adaptive evolution of ruminants in high-altitude regions is closely linked to energy metabolism in the rumen [3]. As a fermentation organ unique to ruminants, the rumen harbors microbial communities that degrade carbohydrates to produce short-chain fatty acids (SCFAs), supplying approximately 75% of the host’s energy requirements [4,5]. In this process, the rumen epithelium serves not only as the primary interface for SCFA absorption but also as a crucial site for their metabolic conversion. Studies have shown that not all absorbed SCFAs enter the systemic circulation; more than 50% are directly metabolized within rumen epithelial cells. Specifically, about 75% of propionate and 95% of butyrate are oxidized and utilized before crossing the epithelium into the bloodstream [6,7]. These metabolic processes not only provide energy for epithelial cells themselves but also participate in the regulation of systemic energy homeostasis through pathways such as ketogenesis [8]. Although previous studies have revealed the transcriptional characteristics of the rumen epithelium during immune barrier formation and development [9] and have achieved considerable understanding of the transmembrane transport mechanisms of SCFAs [10], little is known about the intracellular metabolic fate of SCFAs after entering Tibetan sheep rumen epithelial cells, especially their regulatory mechanisms under high-altitude hypoxia stress.

At the cellular level, cells in the basal and spinous layers of the rumen epithelium are rich in mitochondria, which are the main sites for SCFA oxidative metabolism and ketogenesis [11,12,13]. As the cellular “powerhouses”, mitochondria generate the vast majority of ATP via the tricarboxylic acid (TCA) cycle and oxidative phosphorylation (OXPHOS) to sustain cellular functions [14]. Under stress conditions such as high-altitude hypoxia or nutritional restriction, cells often maintain energy balance by remodeling mitochondrial dynamics (e.g., fusion and fission) and switching metabolic patterns (e.g., enhancing ketogenesis) [15,16]. Accumulating evidence indicates that SCFAs (especially butyrate and acetate) can act as signaling molecules to regulate mitochondrial function by activating pathways such as AMPK, promoting OXPHOS and inhibiting excessive autophagy, thereby maintaining intestinal epithelial homeostasis [17,18]. However, it remains unclear how rumen epithelial cells of high-altitude Tibetan sheep coordinate mitochondrial dynamics through elaborate molecular networks to adapt to hypoxic environments. Post-transcriptional regulation of gene expression is a vital mechanism by which organisms rapidly respond to environmental changes. Among these, miRNAs negatively regulate gene expression by binding to target mRNAs and play key roles in cellular metabolism, immune responses, and stress adaptation. Previous studies found that in Tibetan sheep at different seasons or age stages, significant interactions exist among mRNA/miRNA expression profiles, microbial communities, and metabolites in the rumen epithelium, which are involved in modulating immune barrier function [19]. Furthermore, in tissues such as the heart, Tibetan sheep regulate mitochondrial function via mRNA-miRNA networks centered on the PI3K-Akt, Wnt, and PPAR signaling pathways to cope with hypoxic stress [14]. Nevertheless, no systematic reports have yet described whether specific mRNA-miRNA regulatory networks exist in the rumen epithelium of Tibetan sheep across different high altitudes to coordinate mitochondrial dynamics, SCFA metabolism, and immune adaptation. Therefore, in this study, Tibetan sheep grazing at different altitudes were selected, and their rumen epithelial tissues were subjected to integrated mRNA and miRNA sequencing analysis. The objectives were to: (1) identify differentially expressed mRNAs (DEmRNAs) and miRNAs (DEmiRNAs) in response to altitude gradients; (2) construct DEmiRNA-DEmRNA interaction networks and screen key regulatory factors. The results of this study will fill the knowledge gap regarding post-transcriptional regulation in the rumen epithelium of Tibetan sheep and provide a new theoretical basis for revealing the molecular mechanisms underlying ruminant adaptation to high-altitude environments.

Compared with previous studies, the innovations of the present study are as follows: This study systematically explored the post-transcriptional regulatory mechanism underlying the response of rumen epithelium to high-altitude hypoxia in Tibetan sheep, filling the research gap in the post-transcriptional regulation of rumen epithelial function in plateau ruminants. Based on integrated DEmiRNA-DEmRNA transcriptome analysis along continuous altitude gradients, this study revealed the dynamic variations and synergistic regulatory patterns of coding RNAs and non-coding RNAs with the intensification of hypoxic stress. Core DEmiRNA–DEmRNA regulatory axes, such as *oar-miR-370-3p/PCK2* and *novel-miR-781*/*PIK3R5*, which coordinate mitochondrial energy metabolism and immune homeostasis, were predicted in this study. These findings provide novel molecular targets for research on high-altitude adaptation in ruminants. The results of this study can clarify the molecular mechanism of post-transcriptional regulation by which the rumen epithelium of Tibetan sheep adapts to high-altitude hypoxic environments, and provide a theoretical basis for the conservation, development and utilization of plateau livestock germplasm resources.

## 2. Materials and Methods

### 2.1. Experimental Location and Time

**Figure 1:**
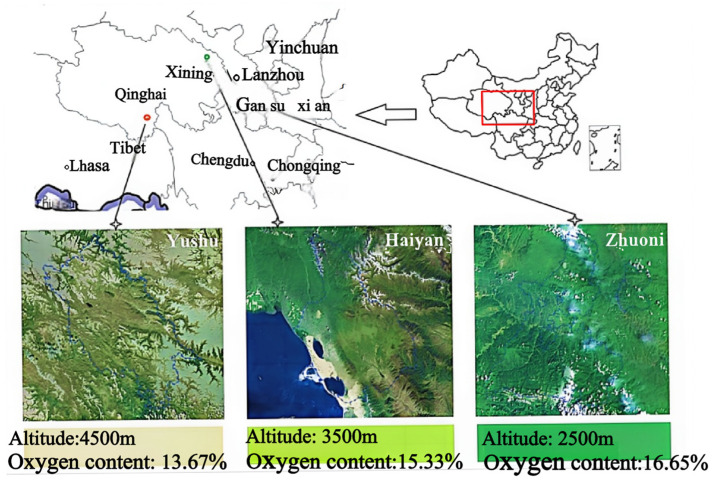
Qinghai–Tibet Plateau sampling sites at three altitudes.

In this study, Tibetan sheep (approximately 3.5 years old, female, 15 individuals) from different altitudinal regions on the Qinghai–Tibet Plateau were selected as experimental animals, including those from Zhuoni (Gansu Province, China) at 2500 m above sea level (TS2500m), Haiyan (Qinghai Province, China) at 3500 m above sea level (TS3500m), and Yushu (Qinghai Province, China) at 4500 m above sea level (TS4500m). Sample collection was conducted during the green grass period in August 2020, and all experimental sheep were freely grazed on alpine grasslands without any supplementary feeding. (The Tibetan sheep used in the experiment were privately owned, and we have obtained the informed consent form from the owner.) The sheep were slaughtered early in the morning before grazing, and rumen ventral sac epithelial tissue samples were collected and rinsed clean with physiological saline; a portion of each sample was then cut and placed into cryotubes, which were immediately stored in liquid nitrogen for subsequent transcriptome sequencing (Figure 1).

### 2.2. Extraction of Total RNA from Rumen Epithelium

Rumen epithelial tissue samples were removed from the −80 °C freezer and immediately ground in a mortar pre-cooled with liquid nitrogen. The ground powdered samples were transferred into 1.5 mL centrifuge tubes, and total RNA was extracted from the rumen epithelium using the Trizol reagent kit (Invitrogen, Carlsbad, CA, USA) following the manufacturer’s instructions strictly. All procedures were performed in a laminar flow hood, and all consumables used were sterile and RNase-free. The extracted RNA was dissolved in DEPC-treated ultrapure water, and RNA concentration and purity were determined using a NanoDrop 2000 spectrophotometer (Thermo Fisher Scientific, Wilmington, DE, USA). A 260 nm/280 nm absorbance ratio between 1.8 and 2.1 indicated that the RNA purity met the experimental requirements. RNA integrity was further evaluated using the Agilent Bioanalyzer 2100 system (Agilent Technologies, Santa Clara, CA, USA).

### 2.3. mRNA Library Preparation and Sequencing (mRNA-Seq)

Sequencing libraries were generated using the mRNA Library Prep Kit for Illumina (Yeasen Biotechnology (China) Co., Ltd., Shanghai, China), strictly following the manufacturer’s instructions. PCR products were purified using the AMPure XP system, and library quality was assessed on the Agilent Bioanalyzer 2100 system. Finally, sequencing was performed on the Illumina NovaSeq platform. Raw reads were further processed for bioinformatic analysis on the BMKCloud online platform (www.biocloud.net) (accessed on 14 April 2026). First, quality control was conducted on raw data to calculate Q20, Q30, GC content, and sequence duplication level of clean data, yielding clean reads. Then, clean reads were aligned to the reference genome (Ovisaries.Oarrambouilletv1.0) using HISAT2. Gene expression levels were quantified by Fragments Per Kilobase of transcript per Million mapped reads (FPKM). Differential expression analysis between two groups was performed using DESeq2, with genes meeting the criteria of Fold Change (|FC|) > 1.5 and *p* < 0.05 defined as DEGs. Furthermore, KEGG pathway enrichment analysis of DEGs was carried out using the KEGG database (http://www.genome.jp/kegg/) (accessed on 14 April 2026), and GO enrichment analysis of DEGs was performed using the GO database (http://www.geneontology.org/) (accessed on 14 April 2026).

### 2.4. miRNA Library Preparation and Sequencing

MiRNA libraries were constructed using the VAHTS™ Small RNA Library Prep Kit (NR801-02). Briefly, 3′ SR and 5′ SR adapters were ligated, followed by reverse transcription to synthesize first-strand cDNA. PCR amplification was then performed, and small RNA libraries were obtained by PAGE gel electrophoresis and gel excision for fragment recovery. PCR products were purified using the AMPure XP system, and library quality was evaluated. Sequencing was performed on the Illumina HiSeq 3500 platform to generate raw reads. Quality control was implemented to remove sequences shorter than 18 nt or longer than 30 nt, trim reads, calculate Q20, Q30, and GC content, and sequence the duplication level of the cleaned data, yielding high-quality clean reads. Clean reads were aligned to the Silva, GtRNAdb, Rfam, and Repbase databases using Bowtie (https://bowtie-bio.sourceforge.net/index.shtml) (accessed on 14 April 2026) to filter out non-coding RNAs (ncRNAs) including rRNA, tRNA, snRNA, snoRNA, and repetitive sequences. Known and novel miRNAs were identified by mapping clean reads to the reference genome (Ovisaries.Oarrambouilletv1.0) and known miRNAs in miRBase. MiRNAs with |FC| > 1.5 and *p* < 0.05 were identified as DEmiRNAs by DESeq2. Target genes of DEmiRNAs were predicted using miRanda (v3.3a) and TargetScan (v5.0). Furthermore, KEGG pathway enrichment analysis of target genes was conducted using the KEGG database (http://www.genome.jp/kegg/) (accessed on 14 April 2026), and GO enrichment analysis of target genes was performed using the GO database (http://www.geneontology.org/) (accessed on 14 April 2026). Multiple testing was corrected using FDR correction, and terms with adjusted *p* < 0.05 were defined as significantly enriched.

### 2.5. Integrated DEmRNA–DEmiRNA Analysis

Based on the mRNA transcriptome sequencing results, via RNAhybrid (v2.1.2) (https://bibiserv.cebitec.uni-bielefeld.de/rnahybrid) (accessed on 14 April 2026) + svmlight (v6.01) (http://svmlight.joachims.org/) (accessed on 14 April 2026), miRanda (v3.3a), (https://cyverse.atlassian.net/wiki/spaces/DEapps/pages/241881574/miRanda+3.3a) (accessed on 14 April 2026) and TargetScan (v7.0) (https://www.targetscan.org/vert_70/) (accessed on 14 April 2026), TargetScanHuman were jointly used to predict potential target genes of DEmiRNAs. The intersection of target genes predicted by the three software programs was defined as the target genes of DEmiRNAs. Given the potential negative regulatory relationship between mRNAs and miRNAs, the Pearson correlation coefficient (PCC) was applied to evaluate the expression correlation between miRNAs and their predicted target genes. Subsequently, DEmRNA–DEmiRNA pairs were screened with PCC < −0.7 and *p* < 0.05, and the DEmRNA–DEmiRNA co-expression network was constructed using Cytoscape software (v3.6.0) (https://cytoscape.org/) (accessed on 14 April 2026). KEGG functional enrichment analysis of mRNAs in the co-expression network was performed using the DAVID online analysis tool. Multiple testing was corrected using FDR correction, and terms with adjusted *p* < 0.05 were defined as significantly enriched. The DEmRNA-DEmiRNA co-expression network constructed in this study was obtained by integrating and screening all differentially expressed mRNAs and miRNAs from three altitude comparison groups (TS3500m vs. TS4500m, TS2500m vs. TS4500m).

### 2.6. RT-qPCR Validation

All mRNAs and miRNAs selected for validation in this study met the criteria of significant differential expression (|FC| ≥ 1.5, *p* < 0.05). RNA samples with qualified quality and concentration were used as templates to synthesize first-strand cDNA according to the instructions of the reverse transcription kit. Using the synthesized cDNA as a template, RT-qPCR was performed on a Roche LightCycler 96 (Basel, Switzerland) real-time fluorescence quantitative PCR instrument to quantify the expression levels of selected RNA molecules at each altitude. *β-actin* primers were used as internal reference genes to normalize the expression levels of mRNAs and miRNAs, respectively. A two-step amplification procedure was used: 95 °C for 30 s; 40 cycles of 95 °C for 5 s; and 60 °C for 30 s. The reaction mixture was as follows: 1 μL cDNA, 0.4 μL each of 10 μmol/L forward and reverse primers (Table 1), 10 μL 2× SYBR Green Pro Taq HS Premix (Accurate Biology, Changsha, China), and RNase-free water added to a final volume of 20 μL. Each independent experiment was repeated at least three times. The relative expression levels were calculated using the 2^−ΔΔCt^ [20] method. Primer information is shown in Table 1.

### 2.7. Data Analysis

Statistical analysis of relative gene expression was performed using one-way analysis of variance (ANOVA) with IBM SPSS Statistics 25 software. A *p* < 0.05 was considered statistically significant. Weighted gene co-expression network analysis (WGCNA) [21] was used to analyze the correlation between metabolites and phenotypic values.

## 3. Results

### 3.1. Analysis of mRNA Expression Profiles in the Rumen Epithelium of Tibetan Sheep at Different Altitudes

**Figure 2:**
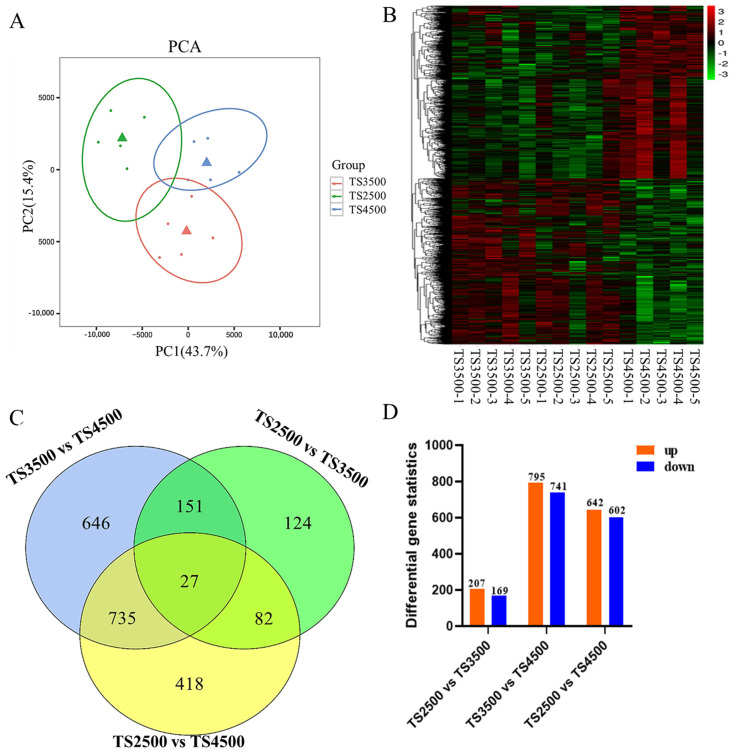
Transcriptome characteristics and differential gene expression analysis of rumen epithelium in Tibetan sheep at different altitudes. (**A**) PCA of gene expression profiles among three altitude groups (TS2500m, TS3500m, TS4500m). (**B**) Heatmap showing the expression patterns of all DEGs across samples from different altitudes. Red indicates upregulated gene expression, and green indicates downregulated gene expression. (**C**) Venn diagram illustrating the overlap of DEGs identified from three pairwise comparisons (TS2500m vs. TS3500m, TS3500m vs. TS4500m, TS2500m vs. TS4500m). (**D**) Statistical histogram of up- and downregulated DEGs in each pairwise altitude comparison. Orange bars represent upregulated genes, and blue bars represent downregulated genes.

Transcriptome analysis was performed on rumen epithelial tissues from 15 Tibetan sheep at different altitudes. A total of 221.48 million clean reads were obtained, and the Q30 of each sample was ≥92.23%. Clean reads from each sample were aligned to the specified reference genome, resulting in mapping efficiencies ranging from 94.03% to 95.08% (Table 2). PCA and hierarchical clustering clearly separated the three altitude groups (Figure 2A), demonstrating that gene expression patterns in the rumen epithelium differed significantly with increasing altitude. A total of 27,916 expressed genes were detected across the three altitudinal groups, among which 2183 genes were identified as differentially expressed. Using |FC| ≥ 1.5 and *p* < 0.05 as the screening criteria, 376 DEGs were found in the comparison between TS2500m vs. TS3500m, including 207 upregulated and 169 downregulated genes; 1536 DEGs were identified between TS3500m vs. TS4500m, including 795 upregulated and 741 downregulated genes; and 1244 DEGs were detected between TS2500m vs. TS4500m, including 642 upregulated and 602 downregulated genes (Figure 2C,D).

**Figure 3:**
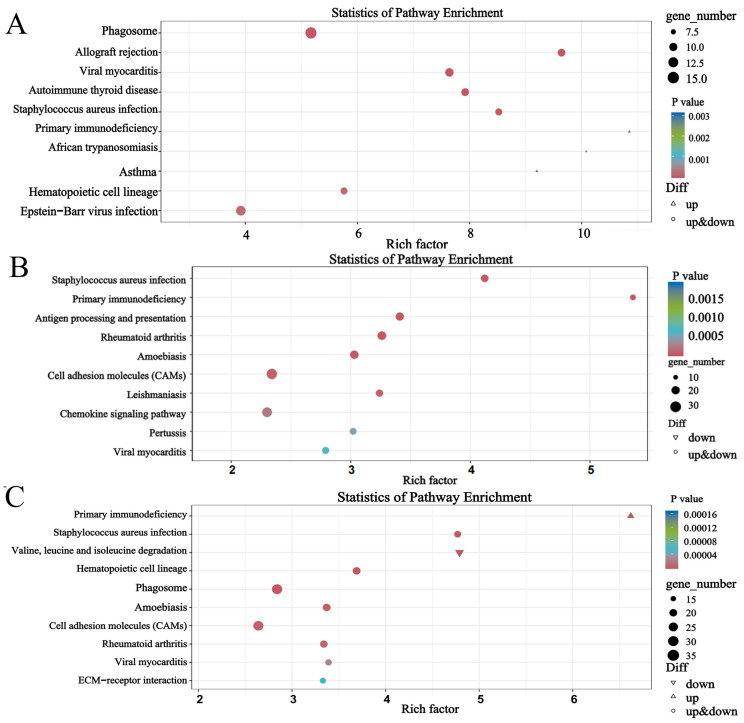

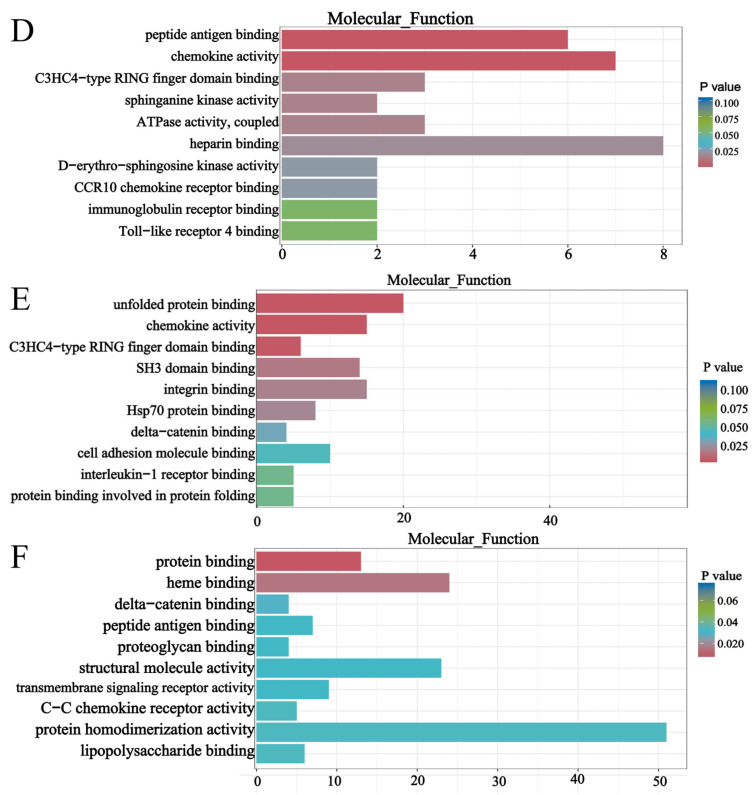
KEGG pathway and GO molecular function enrichment analysis (adjusted *p* < 0.05) of DEGs in ruminal epithelium of Tibetan sheep at different altitudes. KEGG pathway enrichment in (**A**) TS2500m vs. TS3500m; (**B**) TS3500m vs. TS4500m; (**C**) TS2500m vs. TS4500m. GO molecular function enrichment in (**D**) TS2500m vs. TS3500m; (**E**) TS3500m vs. TS4500m; (**F**) TS2500m vs. TS4500m. Only the top 10 enriched pathways are presented in this figure, and other pathways are listed in Attached Figure 1.

Further KEGG functional annotation analysis of the DEGs (Figure 3A–C) revealed that DEGs in the TS2500m vs. TS3500m comparison were mainly enriched in phagosome, Primary immunodeficiency, Epstein–Barr virus infection, NF-kB signaling pathway, and cytokine–cytokine receptor interaction; DEGs in the TS3500m vs. TS4500m comparison were mainly enriched in antigen processing and presentation, cell adhesion molecules (CAMs), chemokine signaling pathway, intestinal immune network for IgA production, phagosome, and cytokine–cytokine receptor interaction; and DEGs in the TS2500m vs. TS4500m comparison were mainly enriched in valine, leucine and isoleucine degradation, phagosome, CAMs, cytokine–cytokine receptor interaction, and NF-kB signaling pathway. Molecular function analysis under the GO classification of DEGs (Figure 3D–F) showed that DEGs in TS2500m vs. TS3500m were mainly enriched in peptide antigen binding, chemokine activity, coupled ATPase activity, heat shock protein binding, and oxidoreductase activity; DEGs in TS3500m vs. TS4500m were mainly enriched in unfolded protein binding, chemokine activity, heat shock protein 70 (Hsp70) protein binding, NADPH quinone reductase activity, coupled ATPase activity, and protein homodimerization activity; and DEGs in TS2500m vs. TS4500m were mainly enriched in protein binding, protein homodimerization activity, and oxidoreductase activity.

### 3.2. Analysis of miRNA Expression Profiles in the Rumen Epithelium of Tibetan Sheep at Different Altitudes

**Figure 4:**
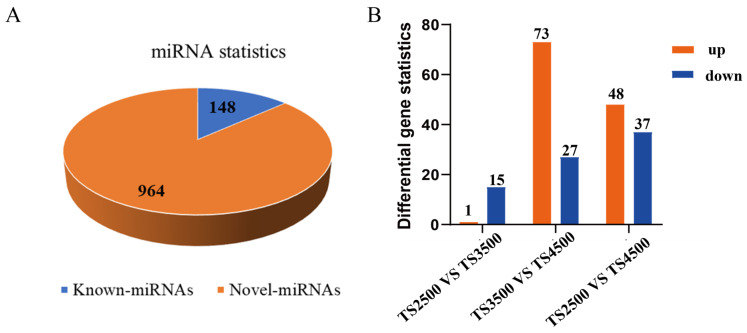
miRNA statistics in rumen epithelium of Tibetan sheep at different altitudes. (**A**) Pie chart showing the numbers of known miRNAs (148) and novel miRNAs (964) identified in this study. (**B**) Statistical histogram of up- and downregulated DEmiRNAs in three pairwise altitude comparisons (TS2500m vs. TS3500m, TS3500m vs. TS4500m, TS2500m vs. TS4500m). Orange bars represent upregulated miRNAs, and blue bars represent downregulated miRNAs.

In this study, small RNA sequencing was performed on 15 rumen epithelial tissue samples of Tibetan sheep at different altitudes, yielding a total of 279.76 M clean reads, with Q30 ≥ 85% for all samples (Table 3). A total of 1112 miRNAs were identified, including 148 known miRNAs and 964 newly predicted miRNAs, and 18,673 miRNA target genes were predicted. Further statistics on the length distribution of clean reads revealed that small RNA fragments were mainly distributed at 21–24 nt, among which 24-nt sequences were the most abundant. This pattern conformed to the typical length distribution characteristics of eukaryotic miRNAs, indicating reliable quality of small RNA-sequencing data. In addition, a comprehensive analysis of miRNA expression levels across all samples was performed, and the top 10 most abundant miRNAs were screened out. These included known miRNAs such as *oar-miR-370-3p*, *oar-miR-127*, *oar-miR-148a*, *oar-miR-150*, and *oar-miR-200a*, as well as highly expressed novel miRNAs including *novel-miR-781*. Most of these high-abundance miRNAs are involved in cellular metabolism, immune regulation, and stress response, suggesting their fundamental roles in maintaining ruminal epithelial homeostasis in Tibetan sheep (Data S1). Using |FC| ≥ 1.5 and *p* < 0.05 as the screening criteria, 135 DEmiRNAs were identified in total (Figure 4A,B). Among them, 16 DEmiRNAs were found in TS2500m vs. TS3500m, including one upregulated and 15 downregulated miRNAs; 100 DEmiRNAs were detected in TS3500m vs. TS4500m, including 73 upregulated and 27 downregulated miRNAs; and 85 DEmiRNAs were observed in TS2500m vs. TS4500m, including 48 upregulated and 37 downregulated miRNAs.

**Figure 5:**
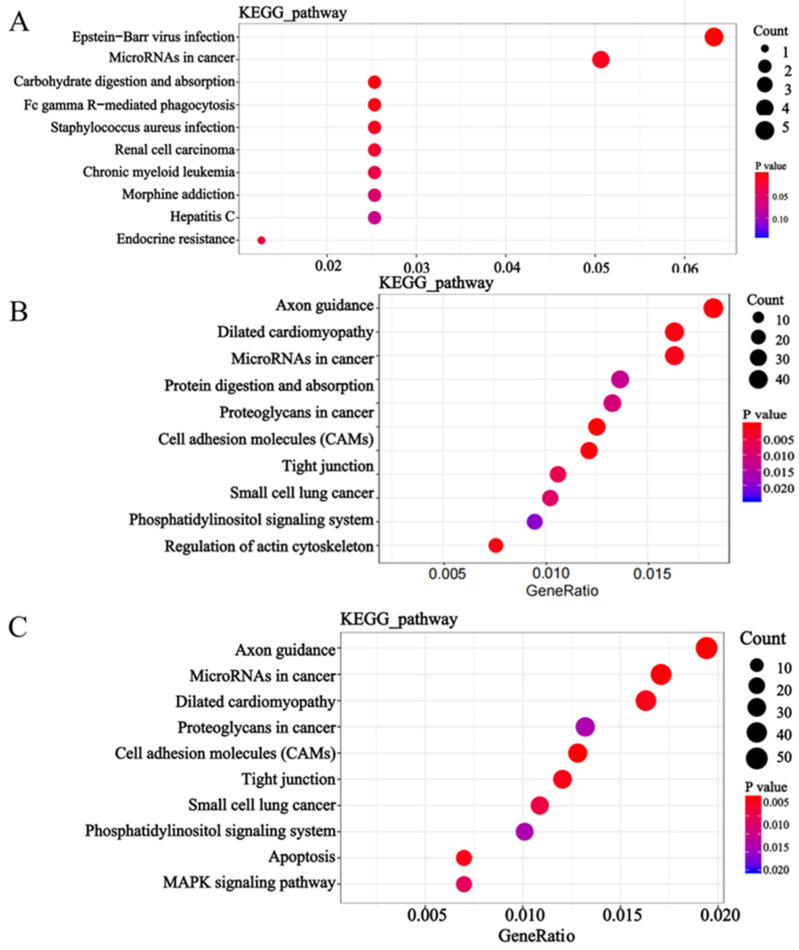

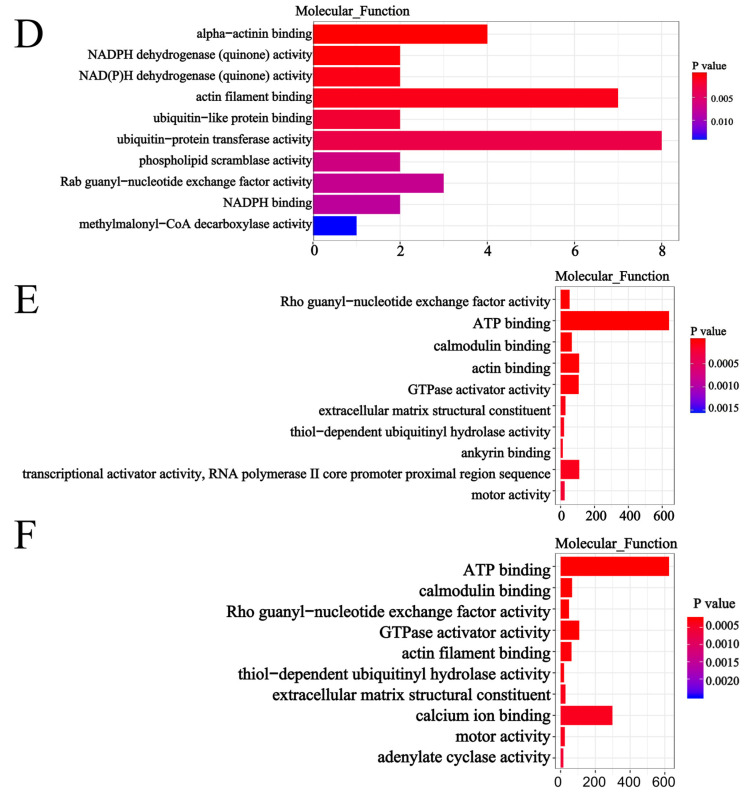
KEGG pathway and GO molecular function enrichment analysis (adjusted *p* < 0.05) of DEmiRNA target genes in rumen epithelium of Tibetan sheep at different altitudes. KEGG pathway enrichment in (**A**) TS2500m vs. TS3500m; (**B**) TS3500m vs. TS4500m; (**C**) TS2500m vs. TS4500m. GO molecular function enrichment in (**D**) TS2500m vs. TS3500m; (**E**) TS3500m vs. TS4500m; (**F**) TS2500m vs. TS4500m. Only the top 10 enriched pathways are presented in this figure, and other pathways are listed in Attached Figure 1.

Further KEGG pathway enrichment analysis of the target genes of miRNAs (Figure 5A–C) revealed that in TS2500m vs. TS3500m, these target genes were mainly enriched in carbohydrate digestion and absorption and Fc gamma R-mediated phagocytosis; in TS3500m vs. TS4500m, they were mainly enriched in CAMs, ABC transporters, axon guidance, tight junction, MAPK signaling pathway, and protein digestion and absorption; and in TS2500m vs. TS4500m, they were mainly enriched in CAMs, axon guidance, tight junction, ABC transporters, Hippo signaling pathway, and MAPK signaling pathway. Further enrichment analysis of molecular functions under the GO classification showed that in TS2500m vs. TS3500m, target genes were mainly enriched in alpha-actinin binding, NADPH dehydrogenase (quinone) activity, and ubiquitin-protein transferase activity; in TS3500m vs. TS4500m, they were mainly enriched in ATP binding, calmodulin binding, GTPase activator activity, and calcium ion binding; and in TS2500m vs. TS4500m, they were mainly enriched in ATP binding, GTPase activator activity, and calcium ion binding (Figure 5D–F).

### 3.3. Analysis of mRNA-miRNA Regulatory Networks in the Rumen Epithelium of Tibetan Sheep at Different Altitudes

**Figure 6:**
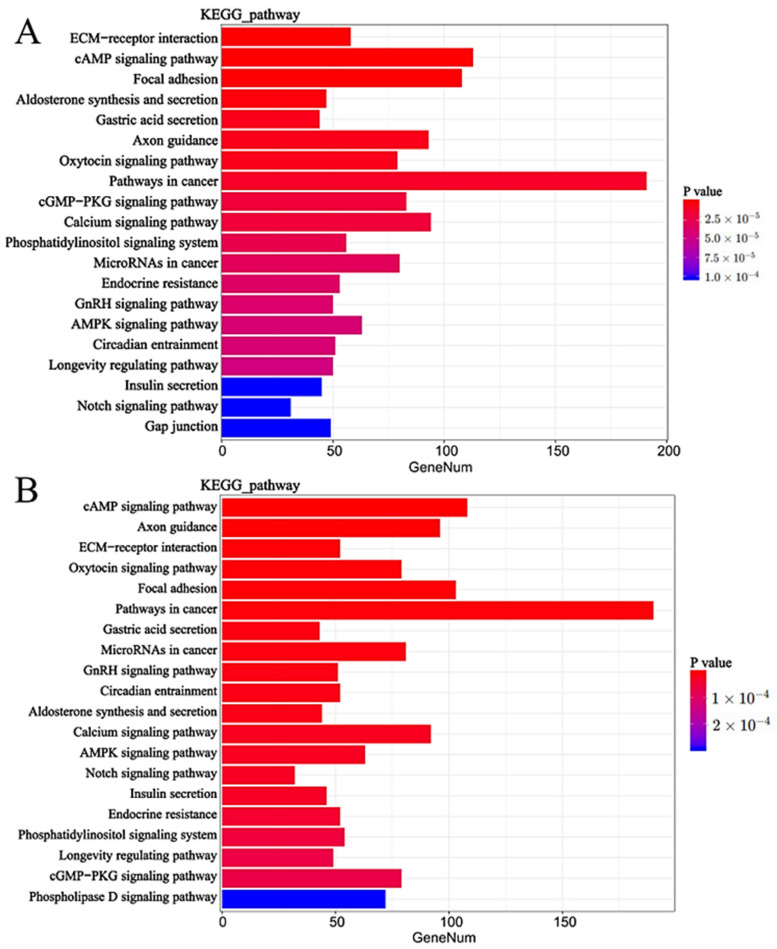
KEGG functional enrichment analysis of target genes in DEmRNA-DEmiRNA regulatory networks in rumen epithelium of Tibetan sheep at different altitudes (adjusted *p* < 0.05). (**A**) KEGG pathway enrichment for the comparison of TS2500m vs. TS4500m; (**B**) KEGG pathway enrichment for the comparison of TS3500m vs. TS4500m.

**Figure 7:**
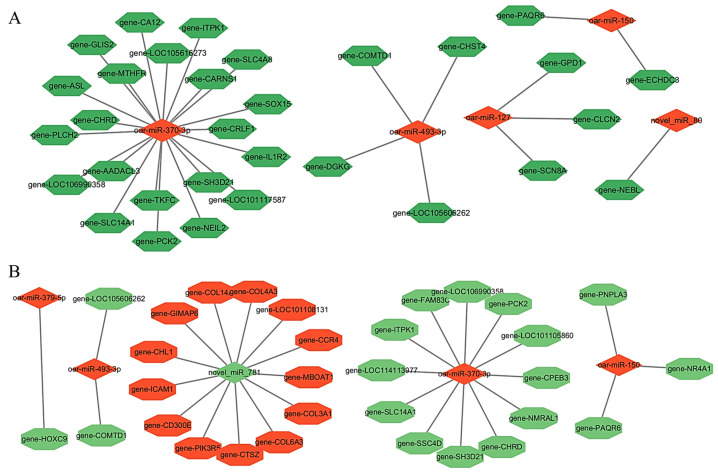
Regulatory network of key DEmiRNA-DEmRNA target pairs in rumen epithelium of Tibetan sheep at different altitudes. No significant regulatory network was detected in TS2500m vs. TS3500m group. (**A**) DEmiRNA-DEmRNA interaction network for the comparison of TS4500m vs. TS2500m; (**B**) DEmiRNA-DEmRNA interaction network for the comparison of TS4500m vs. TS3500m. The relationship pairs were screened with |PCC| > 0.7 and *p* < 0.05. Diamond nodes represent microRNAs (miRNAs), and octagon nodes represent messenger RNAs (mRNAs).

Correlation analysis was performed on differentially expressed DEmRNAs and DEmiRNAs in the rumen epithelium to screen co-expressed DEmRNA-DEmiRNA pairs. Notably, no significant co-expressed DEmRNA-DEmiRNA pairs and enriched KEGG pathways were identified in the TS2500m vs. TS3500m comparison, while valid regulatory pairs were obtained from TS4500m vs. TS2500m and TS4500m vs. TS3500m groups. KEGG functional enrichment analysis of the target genes in the regulatory network (Figure 6A,B, Data S2) revealed that these genes were mainly enriched in pathways including pathways in cancer, cAMP signaling pathway, calcium signaling pathway, AMPK signaling pathway, and focal adhesion. Furthermore, a co-expression network of DEmRNAs and DEmiRNAs was constructed using Cytoscape software (Figure 7A,B), from which a total of 354 pairs of targeted regulatory interactions between DEmRNAs and DEmiRNAs were screened. Several miRNAs, including *oar-miR-370-3p*, *oar-miR-493-3p*, *oar-miR-127*, *oar-miR-150*, and *novel-miR-781*, were found to interact with their target genes. Specifically, the mitochondrial function-related gene *PCK2* was regulated by *oar-miR-370-3p* (FDR = 0.01, log_2_FC = 7.88). *PIK3R5* and the cell morphology-associated gene *COL4A3* were both targeted by *novel-miR-781* (FDR = 0.04, log_2_FC = 6.13). The immune-related gene *IL1R2* was modulated by *oar-miR-370-3p* (FDR = 0.01, log_2_FC = 7.88). In addition, the lipid metabolism and energy production-related gene *GPD1* was regulated by *oar-miR-127* (FDR = 0.04, log_2_FC = 4.52).

### 3.4. RT-qPCR Validation Results

**Figure 8:**
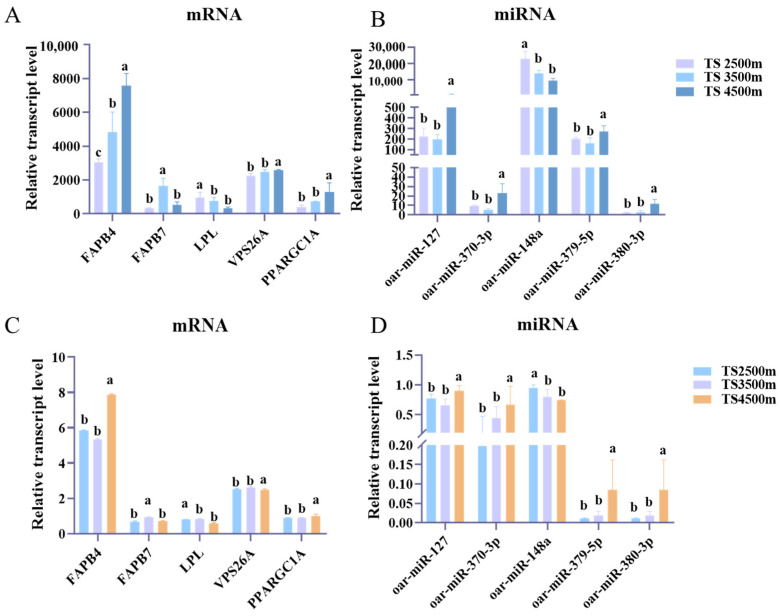
RT-qPCR and RNA-Seq validation. (**A**,**B**) represent the results of RNA-Seq; (**C**,**D**) represent the results of RT-qPCR. Values marked with different superscript letters are significantly different at *p* < 0.05.

Six differentially expressed mRNAs (*FABP4*, *FABP7*, *LPL*, *VPS26A*, *PPARGC1A*) and six DEmiRNAs (*oar-miR-127*, *oar-miR-370-3p*, *oar-miR-148a*, *oar-miR-379-5p*, *oar-miR-380-3p*) were selected for qRT-PCR validation. The results showed that the expression patterns of these mRNAs and miRNAs were consistent with the sequencing data (Figure 8), indicating that the high-throughput sequencing data were reliable.

## 4. Discussion

The rumen is a key organ for nutrient absorption, energy metabolism and immune barrier formation in ruminants, and its functional stability directly determines the adaptability of Tibetan sheep to high-altitude environments. In this study, integrated mRNA-miRNA transcriptome sequencing was used to analyze the rumen epithelial tissues of Tibetan sheep at three altitudes, and the molecular mechanism of rumen epithelium adaptation to high-altitude hypoxia was systematically explored. The main innovations of this study are reflected in three aspects: First, this study focuses on the rumen epithelium, a key functional tissue, and systematically reveals the miRNA-mediated post-transcriptional regulatory network of Tibetan sheep adapting to high-altitude hypoxia, which is a supplement to the existing high-altitude adaptation mechanism literature mainly focused on heart, liver and muscle tissues. Second, based on the continuous altitude gradient design of TS2500m, TS3500m and TS4500m, we clarify the dynamic expression characteristics of coding and non-coding RNAs and the trend of pathway enrichment, which more truly reflects the adaptive process of rumen epithelial function with the increase in altitude. Third, key mRNA-miRNA regulatory axes associated with mitochondrial function and immune response were screened out, which can be used to further explore the core molecular mechanism underlying the balance between energy supply and immune defense in rumen epithelium under hypoxic stress.

As a signature organ of ruminants, the rumen plays a vital role in the metabolism and immune processes of Tibetan sheep, and is closely related to their adaptive evolution to the plateau [22]. In this study, we performed an integrated analysis of mRNA and miRNA expression profiles, mitochondrial structural and functional characteristics, and correlation analysis with microbial metabolites in the rumen epithelium of Tibetan sheep grazing at different altitudes, to explore the regulatory mechanisms underlying immunity and energy metabolism in the rumen epithelium across altitudinal gradients. Transcriptional characteristics of the rumen epithelium are associated with immunity, epithelial development, and metabolism [21]. In the present study, 2183 DEGs were detected in the rumen epithelium of Tibetan sheep at three altitudes, indicating differences in immunity and metabolism among groups. KEGG functional analysis revealed that DEGs were mainly enriched in immune-related pathways including the NF-kB signaling pathway, cytokine–cytokine receptor interaction, and the intestinal immune network for IgA production [23,24]. A previous study reported that the cytokine IL-6 mediates the NF-kB signaling pathway [23]. Among the enriched genes, chemokine-encoding genes *CCL19*, *CCL20*, *CCL21*, and interleukin receptor/ligand genes *IL17RB* and *IL1B* were downregulated at 3500m but upregulated at TS4500m. Upregulation of CCLs may modulate immune functions; for instance, *CCL19* enhances CD8^(+)^ T-cell responses [25], improving immune efficiency and strengthening pathogen defense. Upregulated expression of IL family genes may promote inflammatory responses [26,27]. IgA produced by intestinal epithelial cells is crucial for intestinal immunity [28], and the enriched genes *MADCAM1* and *ITGB7*, which function in the intestinal immune system [29,30], were both upregulated at TS4500m. Their synergistic effect enhances intestinal mucosal immunity, maintains intestinal health, and thereby improves adaptation to harsh high-altitude environments. Comparisons between low and high altitudes showed that several DEGs were enriched in valine, leucine and isoleucine degradation. The catabolism of branched-chain amino acids is critical for energy homeostasis and nutritional metabolism [31], thereby regulating energy metabolism in high-altitude Tibetan sheep. Previous studies reported that rumen epithelial genes participate in energy-producing pathways such as glycolysis, the TCA cycle, and OXPHOS [32]. According to molecular functional analysis of GO classification in this study, differentially expressed genes were mainly enriched in mitochondrial function-related pathways including ATPase activity (coupled), Oxidoreductase activity, Hsp70 protein binding, and NADPH:quinone reductase activity [33,34]. Among them, *HSPA1A* and *HSPA6*, members of the enriched Hsp70 family, were downregulated at TS3500 m and upregulated at TS4500m. Hsp70 plays a vital role in protein folding, unfolding and degradation [35]. In addition, *AIFM3* and *SCCPDH* enriched in oxidoreductase activity are involved in the regulation of apoptosis and mitochondrial function [36], and were downregulated at TS4500m. *SCCPDH*, also known as succinyl-CoA reductase, indicates that the level of mitochondrial oxidative phosphorylation decreases at the high altitude of TS4500m.

Furthermore, our study identified differential miRNA expression in the rumen epithelium of Tibetan sheep across altitudes. Target genes of these DEmiRNAs were mainly enriched in energy metabolism pathways such as carbohydrate digestion and absorption, protein digestion and absorption, as well as immune barrier-related pathways including CAMs, ABC transporters, tight junction, and MAPK signaling pathway [37,38,39], thereby participating in the regulation of rumen epithelial immunity and energy metabolism in Tibetan sheep. GO molecular function enrichment analysis showed that target genes of DEmiRNAs were enriched in mitochondrial oxidative phosphorylation and ATP-related pathways, such as NADPH quinone reductase activity, ATP binding, GTPase activator activity, and calcium ion binding, indicating that DEmiRNAs in the rumen epithelium of high-altitude Tibetan sheep participate in mitochondrial energy metabolism by regulating target genes. Target genes in the regulatory network were mainly enriched in pathways including pathways in cancer, cAMP signaling pathway, calcium signaling pathway, and AMPK signaling pathway, which are associated with mitochondrial energy metabolism and immune processes [40]. Among them, *PCK2*, a target gene related to mitochondrial function, was regulated by *oar-miR-370-3p*. Studies have shown that the pathway involving *PCK2* affects mitochondrial homeostasis [41]. In this study, *PCK2* expression was decreased while *oar-miR-370-3p* was increased in high-altitude Tibetan sheep, suggesting that *PCK2* in the rumen epithelium at TS4500m is regulated by *oar-miR-370-3p*, thereby modulating metabolic homeostasis of epithelial mitochondria. In addition, *PIK3R5* was regulated by *novel-miR-781*. *PIK3R5* has been reported to be mainly enriched in the PI3K-Akt pathway [42], which was also found to harbor mitochondrial function-related DEGs in a transcriptomic study of Tibetan sheep hearts, contributing to high-altitude adaptation [14]. This suggests that *PIK3R5* expression in the rumen epithelium of high-altitude Tibetan sheep is regulated by *novel-miR-781*, thereby modulating epithelial mitochondrial function. Furthermore, *IL1R2* was regulated by *oar-miR-370-3p*. *IL1R2* expression is involved in a functionally specific network linked to IL-1 signaling, NF-kappa B transcription factors, and other complex processes, participating in inflammation [43]. *IL1R2* was highly expressed at TS3500m but decreased at TS4500m, participating in immune regulation in the rumen epithelium of high-altitude Tibetan sheep. We also found that *GPD1*, which is related to lipid metabolism and energy production [44,45], was regulated by *oar-miR-127* and upregulated at TS3500m, indicating enhanced lipid metabolism and energy generation in Tibetan sheep at this altitude. These pathways act synergistically to regulate rumen epithelial function, enabling Tibetan sheep to adapt to harsh high-altitude environments.

## 5. Conclusions

Tibetan sheep employ sophisticated transcriptional and post-transcriptional regulatory strategies in the rumen epithelium to adapt to the harsh high-altitude environment of the Qinghai–Tibet Plateau. Integrated analysis of mRNA and miRNA profiles revealed that with increasing altitude up to TS4500m, Tibetan sheep enhance mucosal immune defense by upregulating chemokines and IgA-related genes, while reshaping the energy metabolism through modulating mitochondrial function and branched-chain amino acid degradation. This study identified key DEmiRNA–DEmRNA interactions, notably the *oar-miR-370-3p*/*PCK2* and *novel-miR-781*/*PIK3R5* axes, which act as critical regulators coordinating mitochondrial homeostasis and immune responses under hypoxia stress. These adaptive mechanisms ensure efficient energy utilization and maintain epithelial barrier integrity, highlighting the rumen epithelium as a central organ in the high-altitude survival strategy of Tibetan sheep. Our findings not only fill the knowledge gap regarding post-transcriptional regulation of digestion in ruminants, but also provide potential molecular targets for breeding livestock with enhanced adaptability to hypoxic and cold environments.

## Figures and Tables

**Table 1:** Information of gene primers.

Genes	Primer Sequence (5′–3′)	Amplicon Length/bp	Accession Number
*FAPB4*	F:GTCCTTCAAATTGGGCCAGG R:TGTACCAGAGCACCTTCATCT	93	NM001114667.1
*FAPB7*	F:AGTACATGAAGGCGCTTGGT R:GTCCCCCTCCTGACTGATGA	89	XM004011152.6
*LPL*	F:CCAAAACTTGTGGCTGCCTTA R:AAACTTGGCCACATCCTGTC	138	NM001009394.1
*VPS26A*	F:TGCCAATGTCCGCTTAAGGT R:AGGTAGCAAGCTGGTGAACA	101	XM027962135.3
*PPARGC1A*	F:GGACTCAAGTGGTGCAGTGA R:GTGAGGACCGCTAGCAAGTT	119	XM015096414.3
*oar-miR-127*	F:atcggatccgtctgagcttggct R: *	-	NR107874.1
*oar-miR-370-3p*	F:gcctgctggggtggaacctggtct R: *	-	NR107887.1
*oar-miR-148a*	F:tcagtgcactacagaactttgt R: *	-	NR107946.1
*oar-miR-379-5p*	F:tggtagactatggaacgtaggc R: *	-	NR107889.1
*oar-miR-380-3p*	F:tatgtaatgtggtccacgtct R: *	-	NR107892.1
*β-actin*	F: AGCCTTCCTTCCTGGGCATGGA R: GGACAGCACCGTGTTGGCGTAGA	113	NM001009784

Note: * The universal reverse primer for RT-qPCR amplification of miRNAs was provided by the Mir-X™ miRNA First-Strand Synthesis Kit.

**Table 2:** Quality control of mRNA sequencing data from 15 rumen epithelial tissues of Tibetan sheep at different altitudes.

Samples	Clean Reads	Clean Bases	GC Content	% ≥ Q30	Mapped Reads
TS2500-1	20,505,404	6,136,767,322	50.55%	92.52%	38,621,362 (94.17%)
TS2500-2	20,907,313	6,257,080,338	50.58%	92.45%	39,556,474 (94.60%)
TS2500-3	22,001,108	6,585,578,932	51.05%	92.55%	41,469,021 (94.24%)
TS2500-4	21,466,009	6,426,557,404	50.62%	92.80%	40,368,803 (94.03%)
TS2500-5	22,650,202	6,785,854,552	50.82%	92.37%	42,911,226 (94.73%)
TS3500-1	22,148,026	6,621,089,810	51.06%	93.16%	41,943,638 (94.69%)
TS3500-2	19,450,345	5,820,575,544	50.85%	92.39%	36,701,424 (94.35%)
TS3500-3	24,469,826	7,325,295,358	50.88%	92.50%	46,405,229 (94.82%)
TS3500-4	23,652,673	7,078,973,074	50.66%	92.50%	44,487,138 (94.04%)
TS3500-5	19,925,847	5,963,392,916	51.19%	92.96%	37,891,319 (95.08%)
TS4500-1	21,138,492	6,321,616,278	50.99%	92.23%	39,975,467 (94.56%)
TS4500-2	20,350,701	6,087,050,230	51.25%	92.90%	38,652,012 (94.96%)
TS4500-3	21,972,750	6,572,412,700	51.03%	92.38%	41,488,687 (94.41%)
TS4500-4	21,846,565	6,535,631,654	51.62%	92.43%	41,300,488 (94.52%)
TS4500-5	19,653,688	5,882,090,704	51.28%	92.37%	37,316,218 (94.93%)

**Table 3:** Quality control of small RNA sequencing data from 15 rumen epithelial tissues of Tibetan sheep at different altitudes.

Samples	Raw Reads	Clean Reads	% ≥ Q30	Mapped Reads
TS2500-1	21,620,747	19,953,758	94.75	7,914,112 (40.41%)
TS2500-2	19,851,432	18,270,457	94.68	8,024,232 (44.61%)
TS2500-3	25,779,874	23,816,056	94.89	9,656,607 (41.09%)
TS2500-4	18,305,063	17,232,556	94.53	6,091,799 (35.95%)
TS2500-5	22,950,967	18,114,790	94.66	7,277,073 (41.71%)
TS3500-1	20,748,128	17,847,428	96.32	7,255,125 (41.39%)
TS3500-2	21,623,364	18,968,918	92.97	7,587,564 (40.86%)
TS3500-3	17,936,243	15,981,079	94.62	5,827,244 (36.82%)
TS3500-4	17,003,774	15,289,164	94.65	6,577,976 (44.47%)
TS3500-5	24,951,726	22,332,434	92.52	9,428,127 (42.89%)
TS4500-1	17,929,488	16,017,222	95.18	7,208,535 (45.88%)
TS4500-2	20,598,067	18,895,257	96.01	8,137,590 (43.53%)
TS4500-3	18,771,962	16,895,709	93.7	7,353,029 (43.91%)
TS4500-4	21,515,801	18,875,285	94.88	8,269,573 (44.40%)
TS4500-5	23,885,389	21,271,593	95.82	9,158,038 (43.47%)

## Data Availability

The data presented in this study are available on request from the corresponding author.

## Supplementary Materials

The following supporting information can be downloaded at: https://www.mdpi.com/article/10.3390/ani16111650/s1, Data S1: Information of miRNA-sequencing data at different altitudes. Data S2: Network construction data of DEmRNAs and DEmiRNAs. Figure S1: KEGG and GO pathway enrichment maps of mRNA and miRNA.
