# Supplementary figures and images for: Integrated mRNA-miRNA Transcriptome Analysis Reveals the Molecular Mechanism of Tibetan Sheep Rumen Epithelium Adaptation to High Altitude
Source: Animals (Basel). 2026 May 28;16(11):1650. doi: 10.3390/ani16111650 (PMC13255973; doi:10.3390/ani16111650)

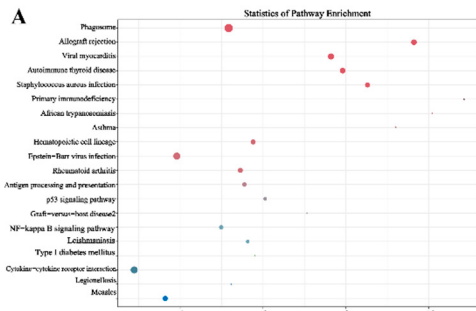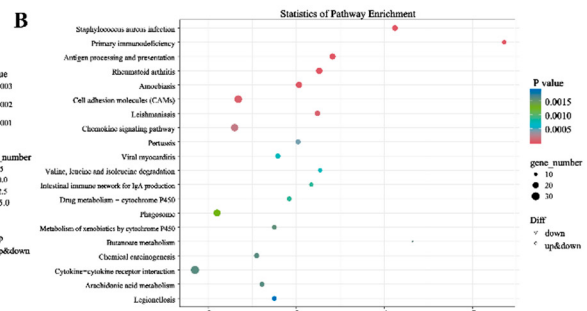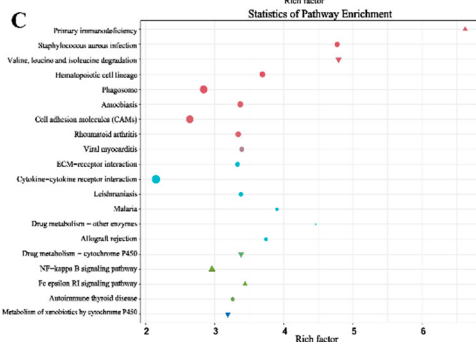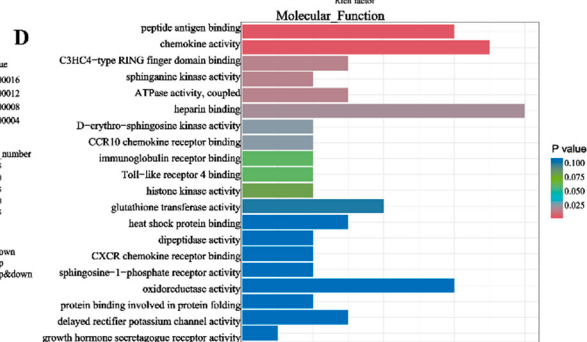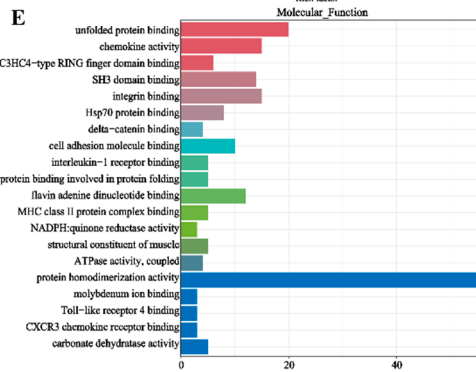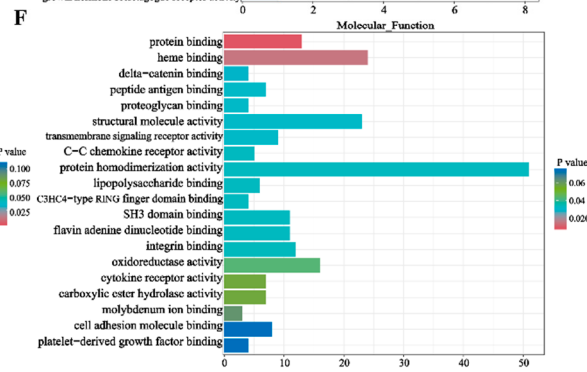

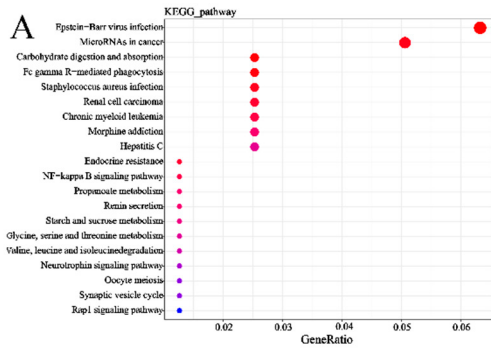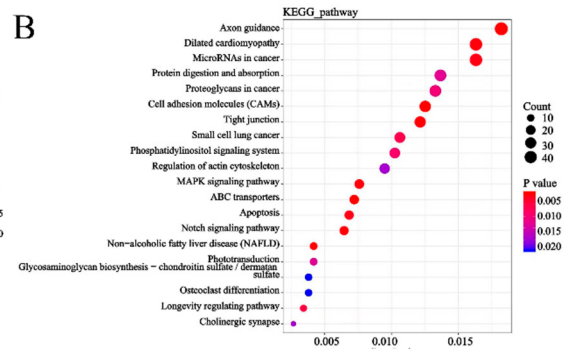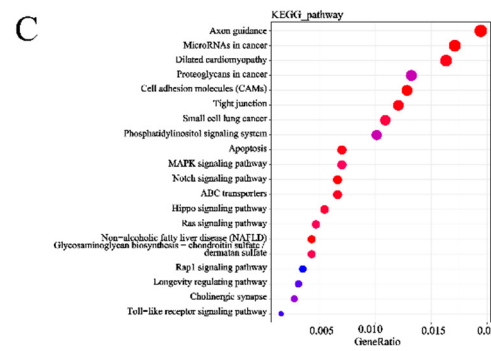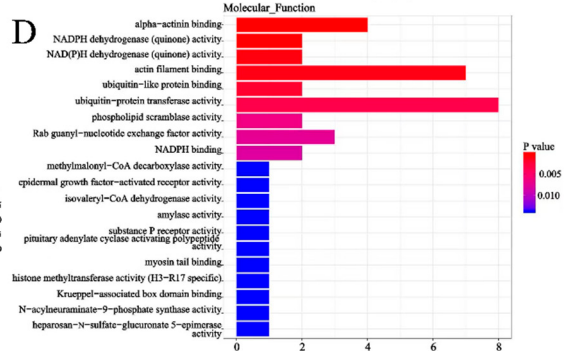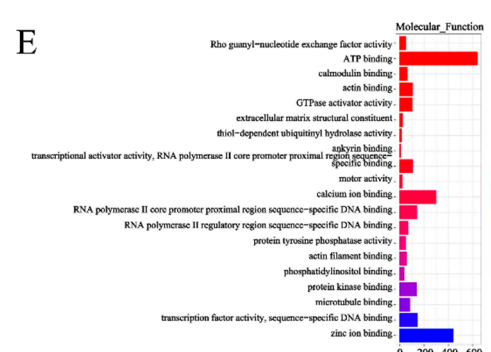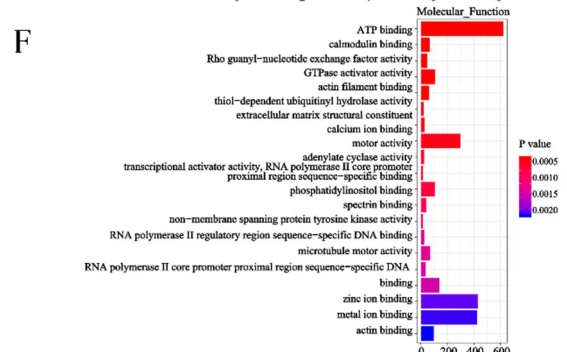

Supplement: Supplementary file 1 [file animals-16-01650-s001.zip › Figure S1.pdf]
